# Radiographic comparison of humeral and glenoid lateralization and distalization between onlay and inlay reverse total shoulder arthroplasty designs in Japanese shoulders

**DOI:** 10.1016/j.jsea.2026.100045

**Published:** 2026-06-06

**Authors:** Eriki Yanagi, Yoshihiro Hirakawa, Tomoya Manaka, Katsumasa Nakazawa, Rei Nishiura, Yoichi Ito, Hidetomi Terai

**Affiliations:** a Department of Orthopaedic Surgery, Osaka Metropolitan University Graduate School of Medicine, Osaka, Japan; b Ishikiriseiki Hospital, Higashi-Osaka, Japan; c Ito Clinic, Osaka Shoulder Center, Matsubara, Japan

**Keywords:** Reverse total shoulder arthroplasty, Lateralization, Distalization, Lateralization shoulder angle, Distalization shoulder angle, Inlay, Onlay

## Abstract

**Background:**

The lateralization shoulder angle (LSA) and modified distalization shoulder angle (mDSA) are radiographic parameters used to assess lateralization and distalization after reverse total shoulder arthroplasty. However, studies comparing implant designs using these parameters are scarce, particularly in smaller Asian populations. We evaluated post-operative distalization and lateralization in Japanese patients and compared 2 implant types.

**Methods:**

We analyzed 139 shoulders of 139 Japanese patients who underwent reverse total shoulder arthroplasty at a single institution. The Aequalis Ascend Flex (Group A, onlay type; Stryker, USA) and Tornier Perform (Group P, inlay type; Stryker, USA) were compared. Group A used a 145° neck shaft angle stem; Group P used a 135° stem with a 10° insert, yielding a 145° total neck shaft angle. All cases used a 36-mm glenosphere with an increased bony offset. Post-operative radiographs measured LSA, mDSA, and Schippers indices: glenoid lateralization angle/humeral lateralization angle and glenoid distalization angle/humeral distalization angle. Stem alignment was assessed, and correlations with range of motion and clinical scores (Constant and American Shoulder and Elbow Surgeons scores) were analyzed.

**Results:**

Groups A and P included 98 and 41 shoulders, respectively. LSA (79 ± 7.2° vs. 85 ± 9.2°) and humeral lateralization angle (26 ± 5.2° vs. 32 ± 6.5°) were significantly higher in Group P (*P* < .001), indicating greater humeral and overall lateralization. Distalization indices (mDSA, humeral distalization angle, glenoid distalization angle) and glenoid lateralization angle showed no significant differences. Approximately 60% of stems were neutrally aligned, though varus alignment was more common in Group P.

**Conclusion:**

Inlay implants demonstrated greater lateralization parameters, mainly due to increased humeral-side lateralization parameters. Implant design and alignment should be considered when interpreting post-operative geometry, especially in smaller Asian/Japanese shoulders.

Reverse total shoulder arthroplasty (rTSA) is a surgical treatment option for cuff tear arthropathy and advanced glenohumeral osteoarthritis.^9^^,^^16^ To date, rTSA implants have undergone various design transitions, and discussions continue regarding the indicators and evaluation methods for post-operative distalization and lateralization. Beginning with the Grammont-type implant, which aims to medialize and distalize the rotation center, subsequent designs have sought to improve clinical outcomes through lateralization. Nevertheless, a unified consensus on the optimal degrees of lateralization and distalization is lacking. Lateralization and distalization are essential for achieving favorable clinical outcomes in rTSA.^6^^,^^10^^,^^12^^,^^23^

Nevertheless, excessive lateralization may cause subacromial impingement and increased stress on the acromion, while excessive distalization has been associated with complications such as nerve palsy.^5^^,^^8^^,^^17^^,^^24^

Boutsiadis and Barth proposed and reported the use of the lateralization shoulder angle (LSA) and distalization shoulder angle as radiographic parameters to evaluate lateralization and distalization after rTSA on standard anteroposterior (AP) radiographs. Although the clinical significance of these indices remains unclear,^2^^,^^3^^,^^7^^,^^11^^,^^13^, ^14^, ^15^^,^^18^^,^^22^ they represent practical and straightforward methods for quantifying post-operative lateralization and distalization.

Furthermore, in 2024, Schippers et al^21^ defined the modified distalization shoulder angle (mDSA), and proposed and reported the subdivision of both the LSA and mDSA into humeral and glenoid components: the humeral lateralization angle (HLA)/glenoid lateralization angle (GLA) and humeral distalization angle (HDA)/glenoid distalization angle (GDA). Clinical utility reports on these parameters remain limited; specifically, no studies have evaluated them according to implant type.

Furthermore, several existing studies on these parameters have not included Asian/Japanese populations. Japanese individuals generally exhibit smaller body sizes, narrower deltoid wrapping angles, and distinct humeral and glenoid morphologies compared with Western individuals, which may affect implant positioning, stem alignment, and post-operative geometry following rTSA. Therefore, evaluating these parameters in Asian populations is crucial for determining their applicability and clinical significance in non-Western cohorts.

This study aimed to assess post-operative distalization and lateralization following rTSA using standard AP radiographs and to compare these parameters across different implant designs, particularly among Japanese individuals. We hypothesized that onlay-type implants would demonstrate greater lateralization values consistent with their design concept.

## Materials and methods

### Patient selection

Between 2018 and 2024, patients who underwent rTSA for cuff tear arthropathy, massive rotator cuff tears (RCT), osteoarthritis (OA), and OA + RCT were retrospectively reviewed. We utilized data from the Shoulder Association of Multi-unit with Rotator Cuff and Arthritis Investigation Database (SAMURAI database), a multi-center collaborative shoulder disorder database constructed on the globally standardized medical patient data collection system, Research Electronic Data Capture (REDCap, http://project-redcap.org, Vanderbilt University, USA). All the patients in the study were Japanese.

All surgeries were performed at a single institution using the same surgical instruments and following the same operative protocol. The onlay-type implant was Ascend Flex (Stryker, USA) (group A), and the inlay type implant was Tornier Perform (Stryker, USA) (group P). Group A used a stem with a 145° neck shaft angle (NSA). Group P used a 135° NSA stem plus a 10° insert (all 0 mm inserts), yielding a total NSA of 145°. Thus, NSA was standardized at 145° in both groups. All procedures included a 5 mm increase in the bony offset (BIO) on the glenoid side, utilizing a 36-mm glenosphere. A 25 mm baseplate was used, with its inferior edge aligned to the inferior glenoid rim and implanted at a 10° inferior tilt. Patient-specific instrumentation (PSI) was used for baseplate placement in all cases. Patients who underwent intraoperative tuberoplasty, defined as resection of the normal greater tuberosity using a bone saw, were excluded.

### Clinical and radiographic evaluation

Patient demographic data, including age at surgery, sex, height, Favard–Walch classification, and diagnosis, were obtained from the SAMURAI database. The following clinical data were collected pre-operatively and 1 year post-operatively: Constant score, American Shoulder and Elbow Surgeons score, and active range of motion of flexion, abduction, external rotation, and internal rotation. Internal rotation was measured by assessing the extent to which the patient could reach behind the back with the thumb and was evaluated on a 6-point scale using the Constant IR score.

**Figure 1:**
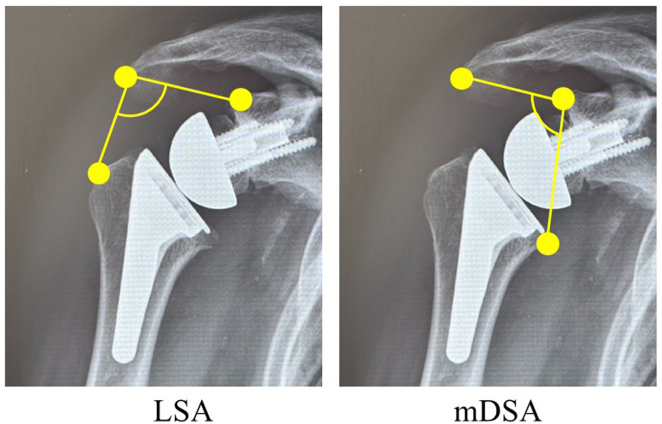
LSA and mDSA. The lateralization shoulder angle (LSA) indicates the angle between the line connecting the superior glenoid tubercle and the most lateral border of the acromion, and the line connecting the most lateral border of the acromion and the most lateral border of the greater tuberosity. The modified distalization shoulder angle (mDSA) is formed by the angle between the line connecting the most lateral border of the acromion and the superior glenoid tubercle, and the line connecting the superior glenoid tubercle and the medial end of the humeral osteotomy.

**Figure 2:**
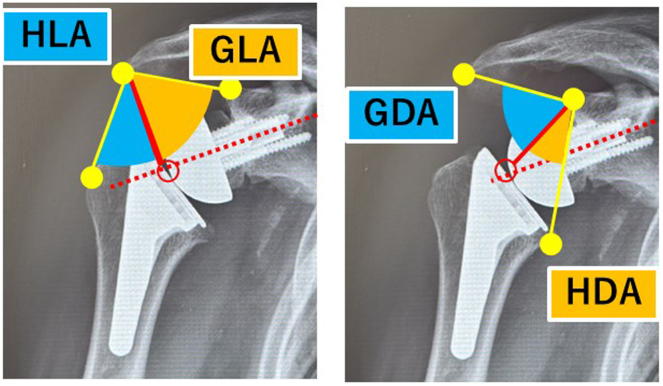
HLA/GLA and HDA/GDA. Based on the glenoid pivot point, the lateralization shoulder angle (LSA) is subdivided into the glenoid lateralization angle (GLA) and humeral lateralization angle (HLA), such that LSA = GLA + HLA. Analogously, the modified distalization shoulder angle(mDSA) is subdivided into the glenoid distalization angle (GDA) and humeral distalization angle (HDA), such that mDSA = GDA + HDA.

**Figure 3:**
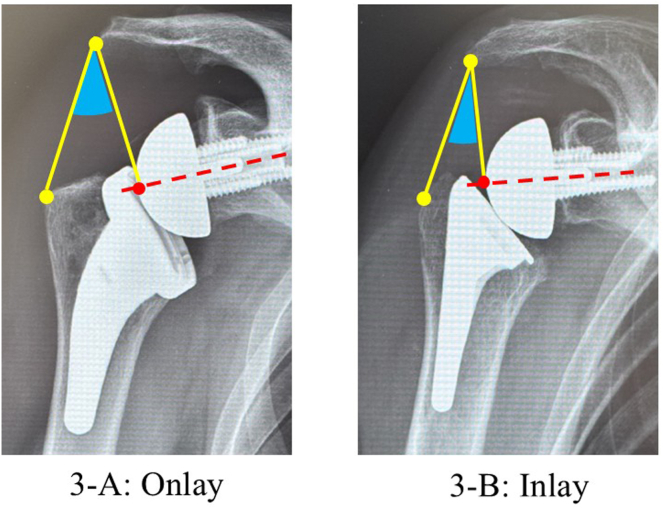
Comparison of HLA across implant types. Plain radiographs after rTSA are shown for both the onlay and inlay types. The humeral lateralization angle (HLA), indicated by the yellow line and blue-shaded area, is larger in the onlay-type. *rTSA*, reverse total shoulder arthroplasty.

Based on the study by Boutsiadis et al,^4^ the evaluation used standard AP radiographs in which the anterior and posterior scapular borders overlapped, constituting a “true AP view”. The limb position was neutral. Radiographs were obtained for 1 year post-operatively. Furthermore, following Schippers et al,^20^ the most lateral point of the glenosphere—the “glenoid pivot point”—served as the reference landmark to divide both the LSA and mDSA (Fig. 1) into 2 components, the glenoid side and humeral side. The HLA/GLA and HDA/GDA were measured and evaluated. Accordingly, the relationships LSA = HLA + GLA and mDSA = HDA + GDA were established, as shown in Figure 2. Figure 3 provides an illustration of the hypothesis presented in the introduction. In the case where the onlay-type implant was inserted (Fig. 3*A*), humeral lateralization—represented by HLA—is significantly greater compared to the case using the inlay type implant (Fig. 3*B*). If the HLA in the onlay-type implant is indeed greater, this finding may be regarded as being in accordance with the fundamental concept and design rationale of the implant.^21^

**Figure 4:**
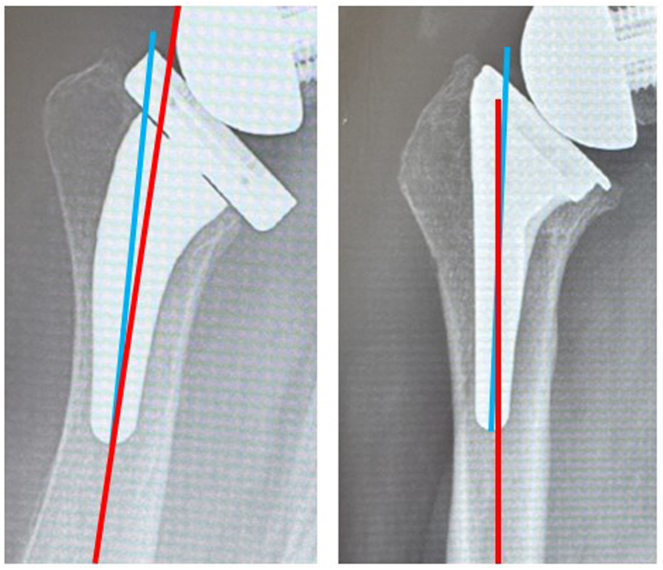
Measurement method for stem alignment. The stem axis is indicated by the blue line, and the humeral shaft axis is indicated by the red line. The angle between these 2 axes is measured, with a range of −5° to 5° defined as neutral. Varus alignment was expressed as a positive value.

Stem alignment was assessed by measuring the angle between the stem and humeral shaft axes based on previously published methods^1^^,^^19^ (Fig. 4). Varus alignment was defined as positive, and angles ranging from −5° to +5° were classified as neutral.

### Statistical analysis

Statistical significance was defined as *P* < .05. All statistical analyses were performed using R (version 3.6.1; R Foundation for Statistical Computing, Vienna, Austria), a graphical user interface designed for medical statistics. Dichotomous variables were assessed using Student *t*-test and chi-square test.

We also calculated the intrarater reliability for all imaging parameters using intraclass correlation coefficients (ICCs). All parameters were measured twice by the same examiner, with an interval of more than two weeks between the 2 sessions.

## Results

### Clinical results

In total, 139 patients who underwent rTSA were included. There were 98 and 41 patients in groups A and P, respectively. Patient demographics are displayed in Table I.

**Table I:** Patient demographic data.

Characteristic	Group A (n = 98)	Group P (n = 41)	*P* value
Sex: male, female	36, 62	22, 19	.09
Age (years)	76 ± 5.8	76 ± 7.5	.85
Height (cm)	154 ± 9.1	157 ± 8.5	.06
Favard classification			.72
E0	82	32	
E1	15	8	
E2	0	1	
E3	1	0	
Walch classification			.30
A1	92	36	
A2	2	3	
B2	2	2	
C	2	0	
Diagnosis			.25
CTA	44	12	
RCT	40	21	
OA	10	7	
OA + RCT	4	1	

*CTA*, cuff tear arthropathy; *RCT*, rotator cuff tear; *OA*, osteoarthritis.

Age and height were compared using the Student *t-*test.

Sex, diagnosis, and Favard and Walch classification were calculated with the chi-test and Fisher exact test.

The details of the implants used are presented in Table II.

**Table II:** Detailed information on the implants used.

Implant	Component	Size	Number
Ascend (Group A)	Tray dial	6	79
		12	19
	Stem size	1B	40
		2B	40
		3B	18
Perform (Group P)	Stem size	1	11
		1+	8
		2	12
		2+	1
		3	9

Table III compares the range of motion and clinical scores before and after surgery, as well as between implant groups. No significant differences were observed between groups pre-operatively.

**Table III:** Pre-operative and post-operative ROM and clinical score.

Parameter	Group A	Group P	*P* value
Pre-Flex (°)	71 ± 43	77 ± 50	.79
Post-Flex (°)	126 ± 27	118 ± 27	.22
*P* value, pre vs. post	<0.01∗	<0.01∗	
Pre-Abd (°)	69 ± 46	78 ± 53	.50
Post-Abd (°)	115 ± 29	105 ± 30	.07
*P* value, pre vs. post	<0.01∗	<0.01∗	
Pre-ER (°)	29 ± 24	35 ± 23	.18
Post-ER (°)	27 ± 20	33 ± 12	.31
*P* value, pre vs. post	0.34	0.28	
Pre-IR	5.5 ± 2.1	5.4 ± 2.2	.11
Post-IR	4.5 ± 2.2	4.8 ± 4.5	.64
*P* value, pre vs. post	0.42	0.15	
Pre-CS	30 ± 7.3	28 ± 22	.43
Post-CS	61 ± 16	61 ± 14	.33
*P* value, pre vs. post	<0.01∗	<0.01∗	
Pre-ASES	45 ± 22	45 ± 25	.86
Post-ASES	77 ± 17	78 ± 19	.64
*P* value, pre vs. post	<0.01∗	<0.01∗	

*Flex*, flexion; *Abd*, abduction; *ER*, external rotation; *IR*, internal rotation; *ROM*, range of motion; *CS*, Constant score; *ASES*, American Shoulder and Elbow Surgeons score.

*P* values were calculated using the Student *t-*test.

∗ Statistically significant (*P* < .05).

Both groups demonstrated significant post-operative improvements in flexion, abduction, Constant score, and American Shoulder and Elbow Surgeons, whereas external and internal rotation did not exhibit significant changes.

### Radiographic results

Intrarater reliability results are shown in Table IV. ICC values were calculated for each implant type, and all groups demonstrated ICCs >0.8, indicating high reliability.

**Table IV:** Intrarater reliability across the 2 measurement sessions.

Parameter	Group	ICC (1,1)	95% Confidence interval
LSA	A	0.88	0.83-0.92
	P	0.93	0.87-0.96
HLA	A	0.95	0.93-0.96
	P	0.97	0.95-0.99
GLA	A	0.85	0.79-0.89
	P	0.85	0.75-0.92
mDSA	A	0.83	0.76-0.89
	P	0.85	0.74-0.92
HDA	A	0.86	0.80-0.90
	P	0.89	0.80-0.94
GDA	A	0.84	0.78-0.90
	P	0.84	0.73-0.91
angle	A	0.80	0.73-0.87
	P	0.90	0.82-0.95

*LSA*, lateralization shoulder angle; *HLA*, humeral lateralization angle; *GLA*, glenoid lateralization angle; *mDSA*, modified distalization shoulder angle; *HDA*, humeral distalization angle; *GDA*, glenoid distalization angle; *ICC*, intraclass correlation coefficient.

ICC (1,1): intrarater reliability between the 2 measurements.

The angle between the stem axis and the humeral axis was defined as positive when in varus.

Table V presents the interimplant comparisons for lateralization and distalization parameters, respectively. For lateralization, both the LSA and HLA were significantly higher in group P; no significant difference was observed in the GLA. For the parameters that showed significant differences (LSA and HLA), the required sample size and statistical power were calculated. For the sample size estimation, the α error was set at 0.05 and the statistical power (1−β error) at 0.80. For the power calculation, the α error was set at 0.05.

**Table V:** Comparison of lateralization and distalization parameters between implant types.

Parameter	Group A	Group P	*P* value
LSA	79 ± 7.2	85 ± 9.2	<.01∗
HLA	26 ± 5.2	32 ± 6.8	<.01∗
GLA	53 ± 5.4	53 ± 5.6	.48
mDSA	103 ± 8.4	103 ± 7.7	.74
HDA	30 ± 7.8	32 ± 6.4	.20
GDA	74 ± 9.5	72 ± 8.0	.18

*LSA*, lateralization shoulder angle; *HLA*, humeral lateralization angle; GLA, glenoid lateralization angle; *mDSA*, modified distalization shoulder angle; *HDA*, humeral distalization angle; *GDA*, glenoid distalization angle.

*P* values were calculated using the Student *t*-test.

∗ Statistically significant (*P* < .05).

The required sample size was 48 shoulders in Group A and 20 shoulders in Group P for the LSA, and 36 shoulders in Group A and 15 shoulders in Group P for the HLA; these requirements were met in the present study. The statistical power was 0.982 for the LSA and 0.998 for the HLA.

For distalization, none of the parameters showed statistically significant differences.

Table VI presents the comparative results for stem alignment. The proportion of neutral alignment was 50% in Group A and approximately 70% in Group P. In group A, all malalignment cases were valgus, whereas in group P, approximately 20% of cases exhibited varus alignment.

**Table VI:** Comparison of stem alignment between implant types.

Parameter	Group A	Group P	*P* value
Angle	−5.0 ± 3.4	1.41 ± 4.5	.01∗
Neutral	49 (50.0%)	29 (70.7%)	.01∗
Valgus	49 (50.0%)	4 (9.8%)	
Varus	0 (0.0%)	8 (19.5%)	

Angle: varus (≥5°), valgus (≤−5°), neutral (−5° < angle <5°).

*P* values were calculated using the Student *t-*test.

∗ Statistically significant (*P* < .05).

## Discussion

This study is the first to compare the newly defined parameters, namely HLA/GLA and HDA/GDA, which separately evaluate lateralization and distalization on the humeral and glenoid sides across implants with different design concepts. Contrary to the initial hypothesis, group P demonstrated significantly greater values for both LSA and HLA than group A. Because LSA is defined as the sum of HLA and GLA, and no significant intergroup difference was observed in GLA, the elevated LSA in group P can be attributed to significantly higher HLA values. Therefore, the humeral-side lateral parameter was markedly greater in group P than in group A, contributing to the overall increase in LSA.

**Figure 5:**
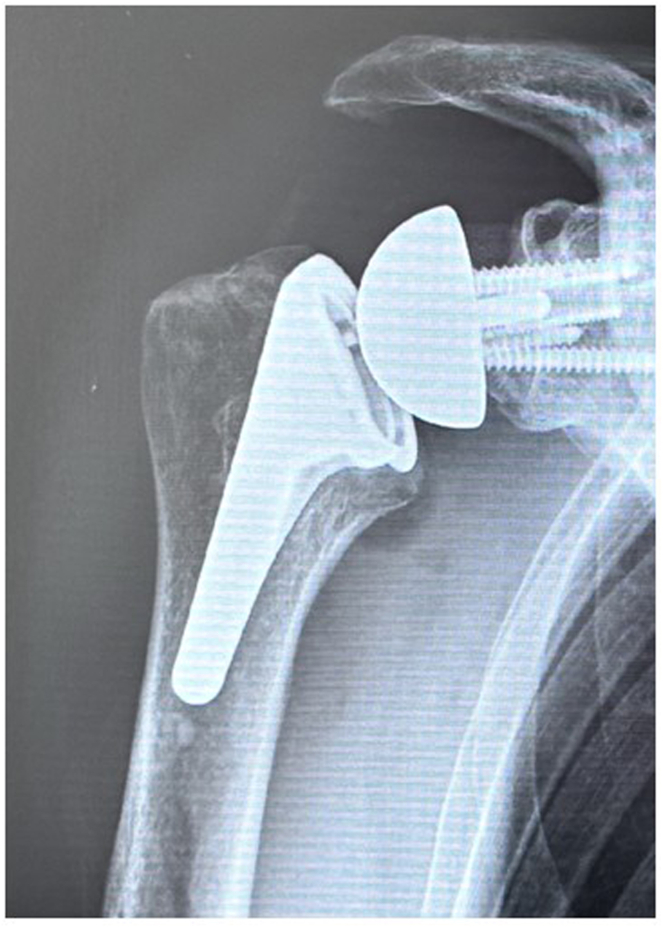
A representative case of varus stem insertion in Group P. The varus stem alignment results in a relative lateralization of the most lateral point of the greater tuberosity.

Several factors may explain the increased HLA levels in the inlay group. First, stem alignment differed between implant types. Here, valgus malalignment accounted for most deviations in group A, whereas approximately 20% of cases in group P exhibited varus alignment. With varus stem insertion, the proximal humerus is relatively laterally displaced. As both LSA and HLA are determined using the most lateral and superior points of the greater tuberosity, this lateral displacement associated with varus alignment likely contributed to the observed increase in the LSA and HLA values. A representative case is illustrated in Figure 5. Although overall alignment in Group P was classified as neutral, the relative increase in varus cases may have elevated these parameters.

One potential explanation for this malalignment is the learning curve associated with adopting a new implant system. Our institution initially used the curved-stem Ascend implant and subsequently transitioned to the Perform implant after its introduction. Proper insertion of the Perform stem requires a sufficiently lateral entry point. However, when surgeons approach the humeral canal with the same technique and mindset used for the Ascend implant, the entry point tends to become relatively medial, which can result in unintended varus alignment. In the present Perform cohort, a substantial proportion of cases were performed during the early phase of implant adoption. This transitional period may have contributed to the higher incidence of varus alignment observed in the inlay group, thereby increasing the humeral-side lateralization parameters.

Differences in the extent of proximal humeral bone resection may also have contributed to the findings. Group A used an onlay-type implant, which generally required more extensive bone removal than did Group P. Consequently, a larger portion of the proximal humerus, including the greater tuberosity, was resected. This alteration of the anatomic landmarks used for plotting may have led to the reduced LSA and HLA values in Group A.

Furthermore, our findings highlight a novel and clinically relevant point: the actual post-operative offset does not always reflect the offset intended by the implant design. Offset values can be substantially altered by variations in stem alignment or the level of proximal humeral resection. Therefore, surgeons should not rely solely on implant-specified offset values but should remain cognizant that both stem malalignment and osteotomy height can meaningfully modify the achieved post-operative geometry.

This study had some limitations. First, the surgeries were performed by multiple surgeons with varying years of experience and technical proficiency. Second, the measurement of the parameters depends on the patient's limb position and posture, which makes it challenging to eliminate the influence of scapular positioning and humeral rotation. Therefore, the parameters and implant alignment may have been underestimated or overestimated. Third, all subjects in this study were Asian (specifically, Japanese). As shown in the demographic data, the average height in both groups was around 150 cm, indicating a relatively small physique. The relatively small physique and unique bone morphology of the Japanese population may have influenced the radiographic findings in this study. Asian patients generally have smaller humeral and glenoid dimensions, narrower deltoid wrapping angles, and thinner cortical bones compared to Western individuals, which may increase the risk of valgus or varus stem alignment depending on the implant design and surgical approach. Specifically, the high proportion of varus alignment observed in the inlay type group may be partly related to the smaller humeral canal and the narrower metaphyseal shape typically observed in Japanese shoulders. Furthermore, these anatomic differences may modulate the relationship between the lateralization parameters and functional outcomes, emphasizing the importance of ethnic and morphological considerations when interpreting radiographic indices after rTSA. Forth, because the extent of bone resection differed between the implant types, the larger resection required in Group A may have prevented accurate plotting of LSA and HLA as originally defined. Fifth, interobserver reliability was not formally assessed in the present study. Although all measurements were performed by a single trained examiner using previously validated protocols with high intrarater reliability (ICC >0.8), the lack of interobserver reliability data remains a limitation. Future studies involving multiple independent examiners are warranted to further confirm the reproducibility of these radiographic indices. Finally, the present results reflect the outcomes of the 2 implant systems evaluated in this study, and therefore, may not be generalizable to other inlay or onlay implant designs.

## Conclusion

Contrary to our initial hypothesis, the humeral lateralization parameters HLA and LSA were significantly greater in the inlay type implant group, which may be due to various factors, including stem alignment, the extent of proximal humeral bone resection, and the relatively small physique of the patients. It is possible that, in Japanese patients, the use of an inlay type implant may not necessarily contribute to medialization depending on the alignment. Keeping this in mind, careful pre-operative planning is essential.

## Disclaimers:

Funding: No funding was disclosed by the authors.

Conflicts of interest: The author, their immediate family, and any research foundation with which they are affiliated have not received any financial payments or other benefits from any commercial entity related to the subject of this article.
